# Supplementary material for: Single-cell dynamics of genome-nucleolus interactions captured by nucleolar laser microdissection (NoLMseq)
Source: Nat Commun. 2025 Dec 17;16:11417. doi: 10.1038/s41467-025-66294-7 (PMC12749836; doi:10.1038/s41467-025-66294-7)
Supplement: Supplementary file 1 — Supplementary Information [file 41467_2025_66294_MOESM1_ESM.pdf]

Supplementary information

Single-cell dynamics of genome-nucleolus interactions captured by nucleolar laser microdissection (NoLMseq)

Kaivalya Walavalkar, Shivani Gupta, Jelena Kresoja-Rakic, Mathieu Raingeval, Chiara Mungo, Philip Rubin, Raffaella Santoro

Extended data Figures 1 to 8

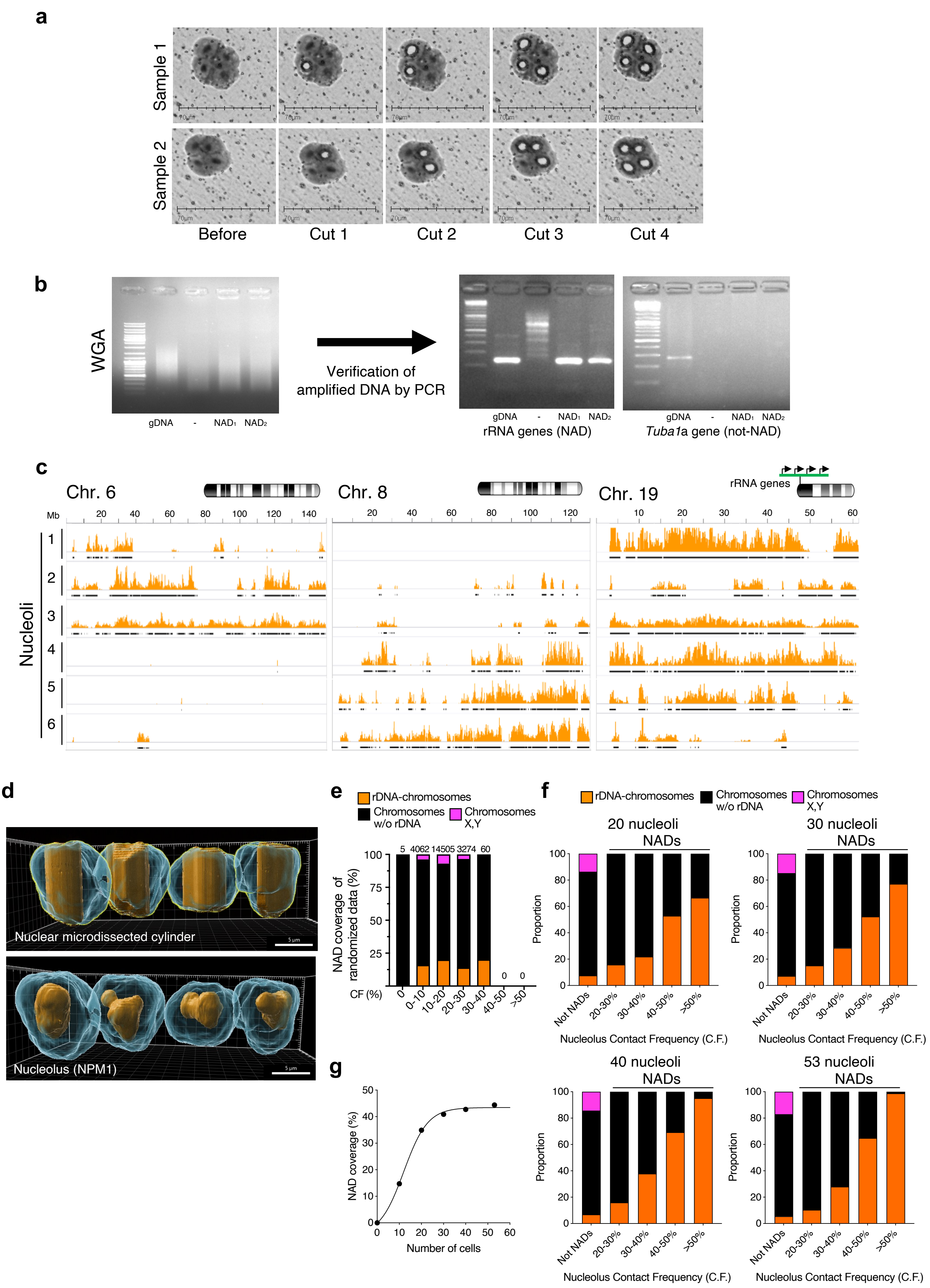

a
Sample 1
Sample 2
Before
Cut 1
Cut 2
Cut 3
Cut 4
b
WGA
gDNA - NAD1 NAD2
Verification of amplified DNA by PCR
rRNA genes (NAD)
Tuba1a gene (not-NAD)
c
Chr. 6
Chr. 8
Chr. 19
rRNA genes
Mb
Nucleoli
d
Nuclear microdissected cylinder
Nucleolus (NPM1)
e
rDNA-chromosomes
Chromosomes w/o rDNA
Chromosomes X,Y
NAD coverage of randomized data (%)
CF (%)
f
20 nucleoli NADs
30 nucleoli NADs
40 nucleoli NADs
53 nucleoli NADs
Proportion
Nucleolus Contact Frequency (C.F.)
g
NAD coverage (%)
Number of cells

Supplementary Figure 1
NoLMseq identifies NADs in single cells
Representative images of ESCs before and after nucleolar laser capture microdissection. **b.** Gel electrophoresis showing whole-genome amplification (WGA) of isolated nucleoli (left panel) and the PCR amplification for rRNA genes and *Tuba1* sequences. **c.** NoLMseq tracks from 6 microdissected nucleoli at chromosomes 6, 8, at 19. Black bars below the tracks indicate NADs called in respective single nucleoli. **d.** 3D image using Imaris showing nuclei (blue) of ESCs with the cylinder representing the microdissection with nucleolus diameter (orange) and nucleoli stained with NPM1 (orange). **e.** Cumulative histogram of genome-wide nucleolar contact frequency (CF) values for randomized sequences for 53 nucleoli matching the genome coverage of each of the 53 ESC nucleoli measured by NoLMseq with respect to their location at chromosomes containing rRNA genes (rDNA-chromosomes), without (w/o rDNA), and X and Y chromosomes. Number indicate the number of 100 kb bins observed at each contact frequency value. **f.** Cumulative histogram of genome-wide nucleolar contact frequency (CF) values in 20, 30, 40 randomly selected microdissected nucleoli and all the 53 analysed nucleoli. The location at chromosomes containing rRNA genes (rDNA-chromosomes), without (non-rDNA chromosomes), and X and Y chromosomes is shown. **g.** NAD coverage calculated from 20, 30, 40 randomly selected microdissected nucleoli and all the 53 analysed nucleoli.
Source data are provided as a Source Data file.

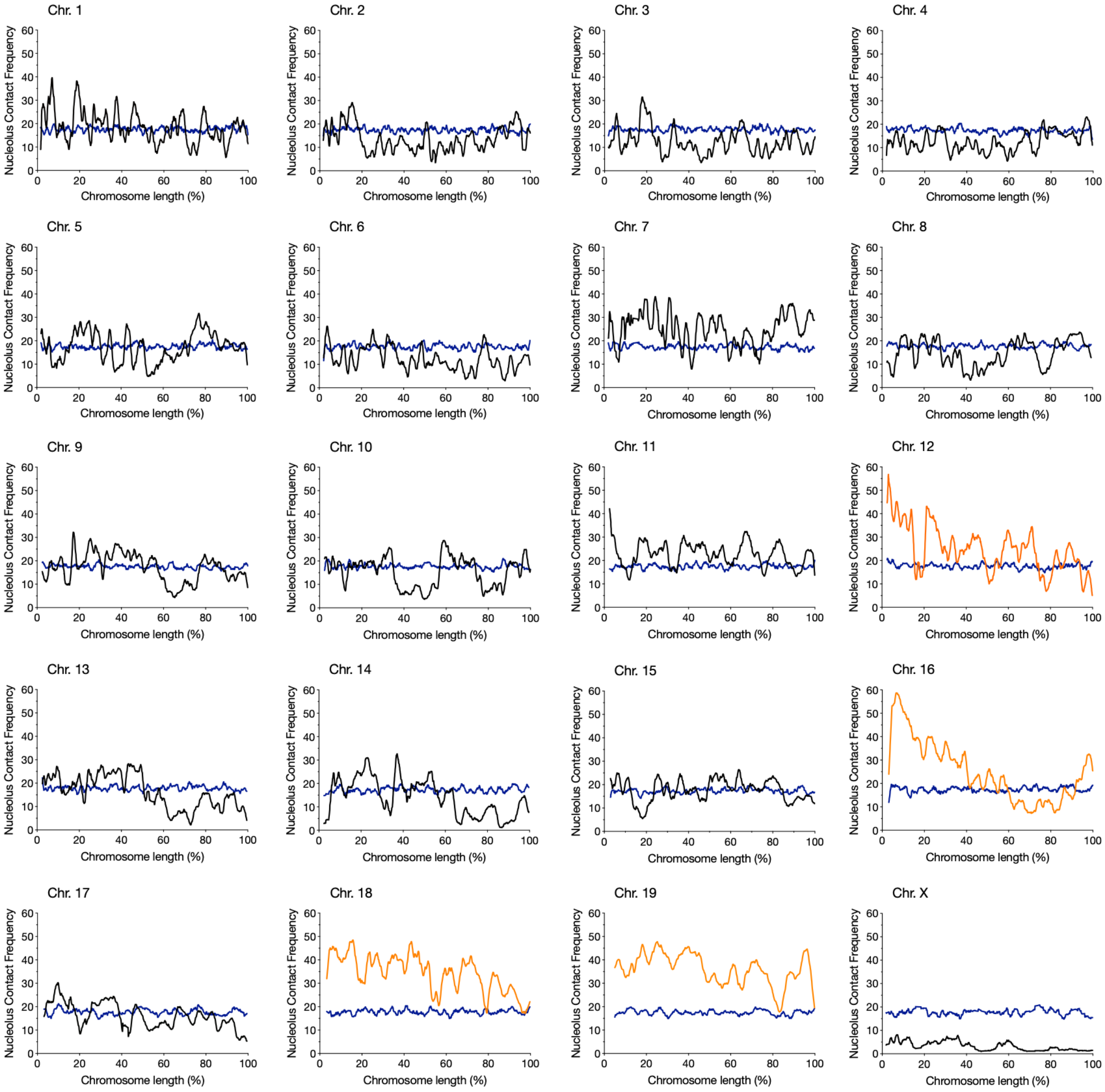

Supplementary Figure 2
NAD contact frequency for each chromosome

Nucleolus contact frequency for the 53 ESC nucleoli along the length of each chromosome. rDNA-chromosomes are depicted with an orange line. Blue line represents CF from 53 randomized samples. Source data are provided as a Source Data file.

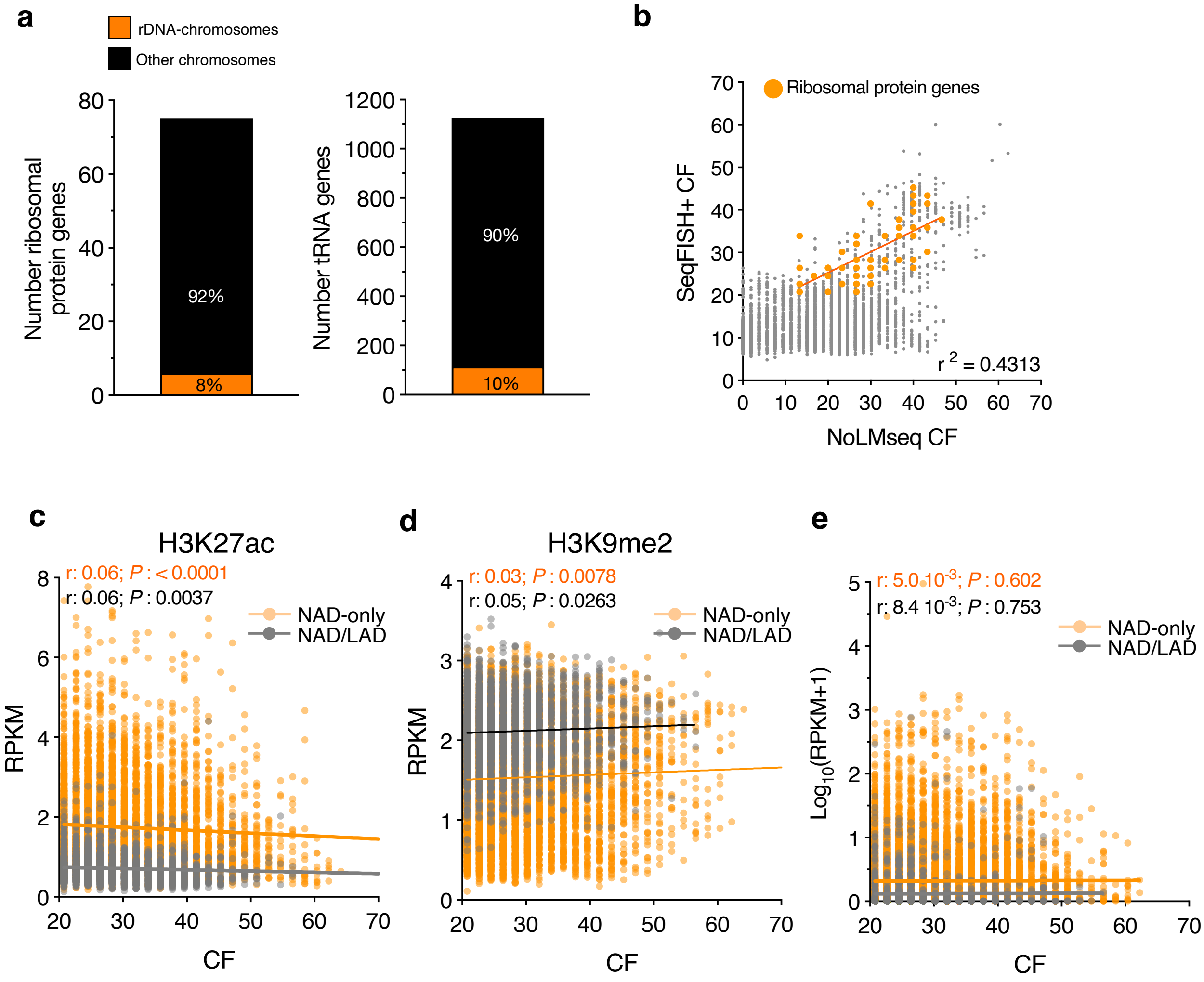

Supplementary Figure 3

Ribosomal protein and tRNA genes show high nucleolar contact frequency

a. Distribution of ribosomal protein and tRNA genes at rDNA-chromosomes and chromosomes not containing rRNA genes (other chromosomes). **b.** Genome-wide comparison of seqFISH+ CF with nucleoli [32] and NoLMseq NAD CF for 3713 paired genomic regions. Ribosomal protein genes are highlighted in orange. **c-e.** Scatter plot showing the levels of H3K27ac (**c**) and H3K9me2 (**d**), and gene expression (**e**) at NAD-only and NAD/LAD as a function of nucleolar CF. r and P values were calculated using simple linear regression. Source data are provided as a Source Data file.

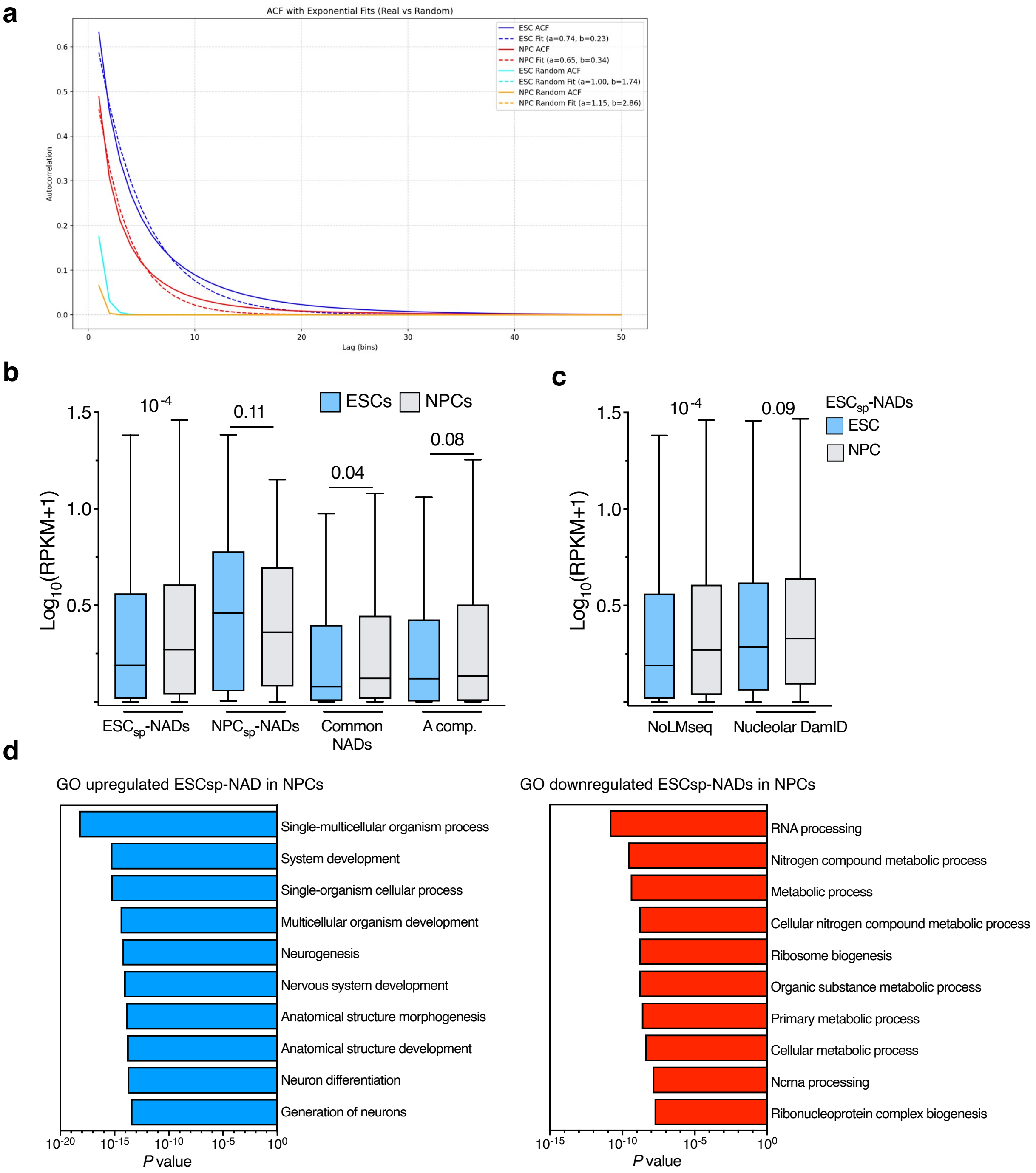

Supplementary Figure 4
NADs from single nucleoli of neural progenitors
a. Autocorrelation values are plotted as a function of increasing lag (in bins), representing the average autocorrelation function (ACF) decay across individual embryonic stem cell (ESC; blue), neural progenitor cell (NPC; red), and ESC (light blue) and NPC (orange) random subpopulations. Dashed lines indicate the fitted exponential decay curves with respective fit parameters. **b.** Gene expression levels (RPKM) levels of genes at ESC_{sp}-NADs, NPC_{sp}-NADs, common NADs, and A compartment. Values are shown as average Log_{10}(RPKM+1). Tukey boxplot where box limits represent the 25th and 75th percentiles. The horizontal line within the boxes represents the median. Statistical significance (*P*-values) was calculated using the two-sided unpaired two-tailed t test. Values with $P > 0.05$ are non-significant. **c.** Comparison of gene expression levels at genes located at ESC_{sp}-NADs detected by NoLMseq and Nucleolar-DamID [20] between ESCs and NPCs. The horizontal line within the boxes represents the median. Statistical significance (*P*-values) was calculated using the unpaired two-tailed t test. Values with $P > 0.05$ are non-significant.**d.** Gene ontology (GO) terms for upregulated and downregulated genes at ESC_{sp}-NADs in NPCs. Source data are provided as a Source Data file.

Histone Mark	Empirical *P* values	Observed *P* values
H3K9me2	0	1.406×10^{-6}
H3K27ac	0	1.128×10^{-23}
H3K4me1	0	9.433×10^{-26}
H3K4me3	0.004	0.0284
H3K9me3	0.752	0.9799
H3K27me3	0.508	0.7985

Supplementary Figure 5
Randomization analysis of NADs
Bootstrapping analysis (n = 1000 iterations) was performed to assess the enrichment of histone modifications between the two embryonic stem cell (ESC) subpopulations. For each mark, the empirical p-value was calculated by comparing the observed enrichment against the distribution from the resampled datasets. Both the empirical P values and the statistical P value of the observed enrichment are indicated.

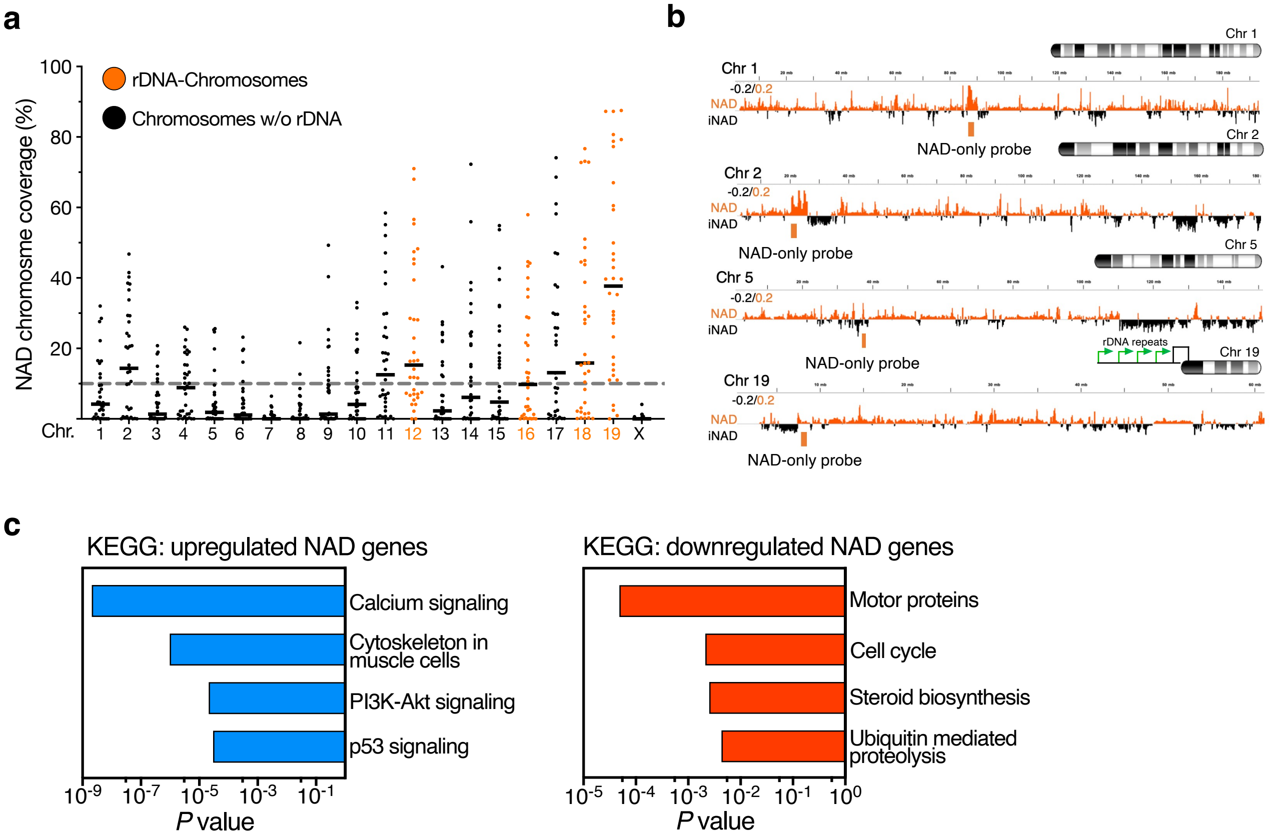

Supplementary Figure 6

Nucleolar integrity is required for genomic contacts with nucleoli

a. Coverage of NADs at each chromosome of single nucleoli from ESCs treated without and with Actinomycin D (ActD). Dotted grey line indicates the average NAD coverage in single cells. Black lines indicate the mean on each chromosome. **b.** Representation of the DNA-FISH probes targeting NADs at chromosomes 1, 2, 5, and 19, the latter containing rRNA genes at the 5' end, close to the centromeric region. The corresponding NADs profiles from Nucleolar-DamID [20] are shown. iNAD: genomic domains not interacting with nucleoli. **c.** KEGG pathways for upregulated and downregulated NAD-genes in ESC+ActD. Source data are provided as a Source Data file.

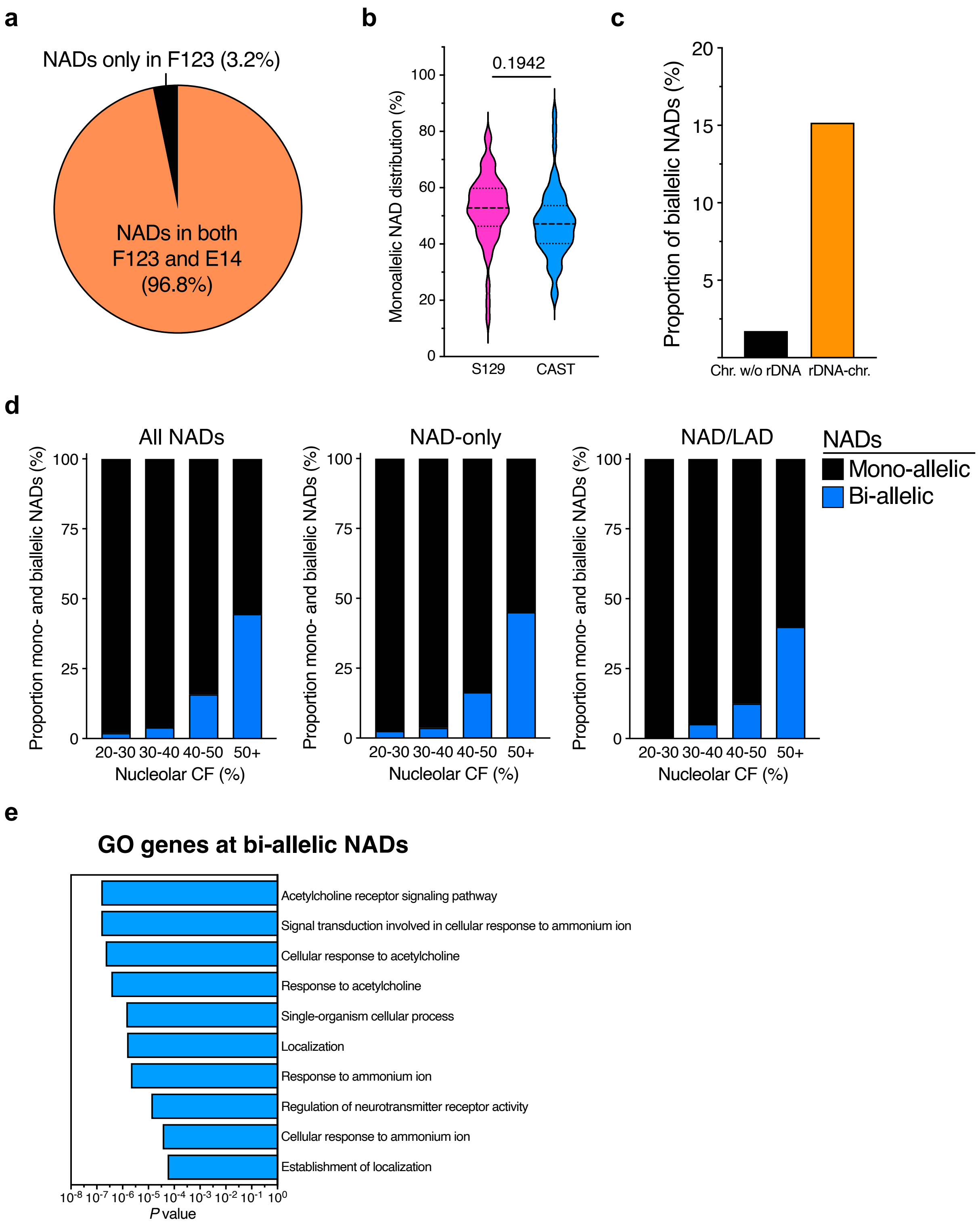

Supplementary Figure 7

Genomic contacts with nucleoli are mono-allelic, but not parent specific.

a. Pie chart showing the proportion of NADs identified in F123-ESCs that have also been found in E14-ESCs. **b.** Mono-allelic distribution of 129 and CAST genome around nucleoli of single cells. Black dashed lines indicate median and black dotted lines indicate quartiles. Statistical significance (P-value) was calculated using the two-sided paired two-tailed t test. **c.** Proportion of NADs from rDNA-chromosomes and non rDNA chromosomes at bi-allelic NADs. **d.** Cumulative histogram of nucleolar contact frequency (CF) values with respect to their mono-and biallelic content for all NADs, NAD-only, and NAD/LAD. **e.** Gene ontology (GO) terms of genes located on bi-allelic NADs. Source data are provided as a Source Data file.

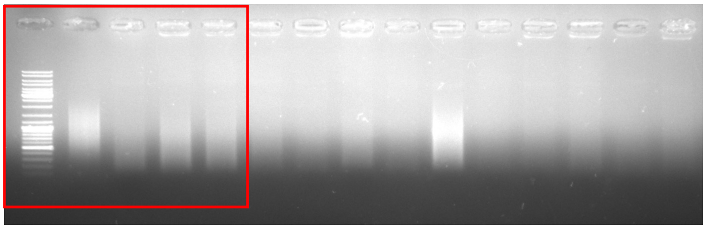

gDNA - NAD_1 NAD_2

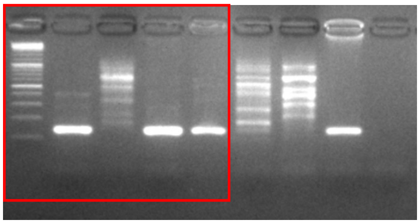

gDNA - NAD_1 NAD_2

rRNA genes (NAD)

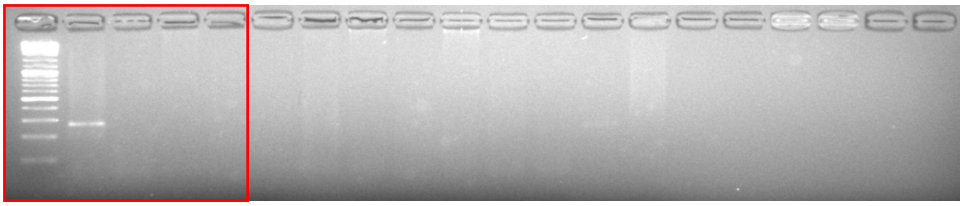

gDNA - NAD_1 NAD_2

*Tuba1*a gene (not-NAD)

Supplementary Figure 8

Uncropped images of gels shown in Supplementary Figure 1b
