# Supplementary material for: Single-cell dynamics of genome-nucleolus interactions captured by nucleolar laser microdissection (NoLMseq)
Source: Nat Commun. 2025 Dec 17;16:11417. doi: 10.1038/s41467-025-66294-7 (PMC12749836; doi:10.1038/s41467-025-66294-7)
Supplement: Supplementary file 2 — Description of Additional Supplementary Files [file 41467_2025_66294_MOESM2_ESM.pdf]

Description of Additional Supplementary Files

Supplementary Data 1: Gene ontology (GO) terms of genes located at ESCsp-NADs.

Supplementary Data 2: Gene ontology (GO) terms, KEGG and Wikipathways analyses of genes located at NADs specific to ESC populations 1 or 2.

Supplementary Data 3: KEGG and Wikipathways analyses of up- and downregulated NAD genes in ESC+ActD.

Supplementary Data 4: List of primers to generate DNA-FISH probes.
